# Immediate and persistent antidepressant-like effects of Chaihu-jia-Longgu-Muli-tang are associated with instantly up-regulated BDNF in the hippocampus of mice

**DOI:** 10.1042/BSR20181539

**Published:** 2019-01-11

**Authors:** Xing Wang, Jie Chen, Hailou Zhang, Zhiheng Huang, Zhilu Zou, Yin Chen, Lei Sheng, Wenda Xue, Juanjuan Tang, Haoxin Wu, Hongquan Liu, Gang Chen

**Affiliations:** 1 Affiliated Hospital of Integrated Traditional Chinese and Western Medicine, Nanjing University of Chinese Medicine; No. 100 Cross Street, Hongshan Road, Nanjing 210028, China; 2 Center for Translational Systems Biology and Neuroscience and Key Laboratory of Integrative Biomedicine for Brain Diseases, College of Basic Medicine, Nanjing University of Chinese Medicine, Nanjing 210023, China; 3 Co-innovation Center of Neuroregeneration, Nantong University, Nantong, Jiangsu 226001, China; 4 Brain Hospital Affiliated to Nanjing Medical University; No. 264, Guangzhou Road, Gulou District, Nanjing 210029, China; 5 School of Pharmacy, Nanjing University of Chinese Medicine, Nanjing 210023, China; 6 The Second Affiliated Hospital of Traditional Chinese Medicine, Nanjing University of Chinese Medicine; No.23 Nanhu Road, Jianye District, Nanjing 210017, China

**Keywords:** BDNF, Chaihu-jia-Longgu-Muli-tang, Immediate and persistent antidepressant-like effects, ketamine

## Abstract

Conventional antidepressants have a disadvantage in delayed onset of efficacy. Here, we aimed to evaluate the immediate and persistent antidepressant-like action of a classic herbal medicine Chaihu-jia-Longgu-Muli decoction (CLM) as well as the action of CLM on hippocampal brain-derived neurotrophic factor (BDNF) over time. CLM consists of Xiaochaihu decoction (XchD), Longgu-Muli (LM) and several other herbs. The contribution of constituent herbal formula XchD and other parts of CLM was also assessed. Following a single dose of CLM, tail suspension test (TST), forced swim test (FST), and novelty-suppressed feeding test (NSF) were performed. The antidepressant activity of XchD, its interaction with LM or remaining parts of CLM was also examined after a single administration. BDNF expression in the hippocampus was examined at 30 min and 24 hr post a single CLM. A single administration of half of clinical dose of CLM elicited antidepressant effects at TST 30 min post administration, and lasted for 72 hr. Furthermore, CLM also reduced the latency to eat in NSF test. A single proportional dose of XchD induced antidepressant effects at 30 min and lasted for 48 hr, whereas the effect lasted for 72 hr when combined with either LM or the remaining parts of CLM. BDNF expression increased at 30 min and persisted at least for 24 hr after a single dose of CLM. The results support that Chaihu-jia-Longgu-Muli decoction was capable to immediately and enduringly elicit antidepressant activity via enhancement of hippocampal BDNF expression, in which the constituent Xiaochaihu decoction played the primary role.

## Introduction

Depression, a widespread incapacitating psychiatric condition, poses a substantial health threat to society [1]. Depression is becoming one of the biggest contributors to disease burden worldwide [2]. Selective serotonin reuptake inhibitors (SSRIs) are the most commonly used antidepressants. However, their antidepressant effects require waiting for several weeks, which is a significant disadvantage to individuals who are particularly vulnerable to suicide [3]. Only approximately two-thirds of patients with depression respond to SSRIs [4]. Additionally, adverse reactions to SSRIs are common, including abnormal gastrointestinal function, sleeplessness, and sexual dysfunction etc [5–7].

Recent studies demonstrated that a single low dose of ketamine produces a rapid antidepressant response that starts as soon as 2 h and may last for several days [8,9]. However, ketamine also exerts psychotomimetic, addictive, and potentially neurotoxic effects, which impose restrictions on its clinical application. Herbal medicine is a major part of traditional Chinese medicine (TCM). Some herbal prescriptions have been shown to elicit and/or enhance the antidepressant responses, and reduce certain side effects associated with psychotropic medications [10–12]. Chaihu-jia-Longgu-Muli-tang (CLM) is one of the most popular herbal formulas in TCM for treatment of mood disorders. CLM has been reported to possess psychotropic actions such as dementia, insomnia, anxiety, stress, and late-onset hypogonadism, and are the prescription of choice for patients with neuropsychiatric disorders [13–16]. Previous studies have reported antidepressant effects of CLM in animal models after chronic administration, and multiple mechanisms have been suggested [17,18]. CLM alleviated the chronic stress-induced depressive-like conditions by preventing the reduction in dopaminergic and serotonergic transmission in the prefrontal cortex, normalizing the dysfunction of the hypothalamo–pituitary–adrenal system [15], and up-regulating the expression of brain-derived neurotrophic factor (BDNF) [17]. However, the rapid antidepressant potential of CLM has not been assessed previously.

Here, the rapid antidepressant-like effects of CLM was evaluated experimentally. Instant and persistent enhancement of neural plasticity via up-regulation of hippocampal BDNF expression is responsible for the rapid antidepressant effect of ketamine, as well as Yueju pill, the first herbal medicine identified to confer rapid antidepressant effects [19,20]. Therefore, hippocampal BDNF was assessed quickly and over time after a single administration of CLM to reveal the plausible mechanisms. The results showed half of daily dose of CLM has rapid antidepressant potential. CLM also induced instant and persistent BDNF up-regulation in the hippocampus. As CLM derived from the prescription of Xiaochaihu decoction (XchD), which itself is implicated in treatment of mood disorders, in combinatiom with Longgu-Muli (LM) and several other herbs, the contribution of XchD and other parts of CLM to the rapid antidepressant-like effects of CLM was also investigated.

## Materials and methods

### Animals

The behavioral experiments were carried out using male BALB/c mice (weight: 20–25 g), purchased from China Academy of Military Medical Sciences (Beijing). Mice aged 6–8 weeks, were habituated to the animal facility for 1 week before behavioral testing. The animals were maintained under standard laboratory conditions (temperature: 22 ± 2°C; humidity: 50 ± 10%) with a 12:12-h light/dark cycle. The animals were fed standard diet and filtered water. The animal experimental procedures were according to the Guide for the Care and Use of Laboratory Animals, and were approved by the Institutional Animal Care and Use Committee at Nanjing University of Chinese Medicine.

### Drugs and decomposed recipes of CLM

The medicinal plants used to prepare CLM were *Radix bupleuri* (Chai HU), Long Gu (Long Gu), *Scutellaria baicalensis* (Huang Qin), Ginger (Sheng Jiang), Ginseng (RenShen), *Cassia* twig (GuiZhi), *Poria cocos* (Fu Ling), *Pinellia ternate* (Ban xia), *Rheum officinale* (Da Huang), *Concha ostreae* (Mu Li), and Fructus Ziziphi Jujubae (Da Zao). All the medicinal plants were purchased from Nanjing GuoYi Clinical (Medicinal Material Department; Nanjing, China). The compositions and chemical constituents of CLM were presented in Table 1. For the preparation of the drugs, each constituent was soaked for 30 min and subsequently mixed with water for 2 h in a 1:8 ratio. Subsequently, the solution was collected, placed in a water bath at 60°C, and evaporated to a target concentration. The amount of raw drugs of CLM in clinical use is 60 g for standard body weight of 60 kg, and thus the clinical dosage is approximately 1 g/kg. The equivalent dose in mice was 10 g/kg. The yield of the preparation was 42%, so the used dose was 4.2 g/kg. The solutions of the herb preparation and vehicle were administered to mice via the intragastric route at a dosage of 0.1 ml/10 g. Ketamine HCl (Gutian Pharmaceuticals, China) was dissolved in saline and administered intraperitoneally.

**Table 1:** Drugs and decomposed recipes of CLM

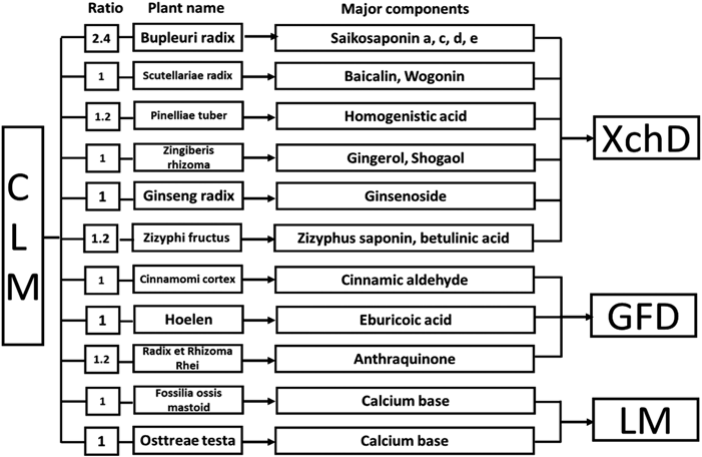

Abbreviations: GFD, Guizhi, Fuling and Dahuang; LM, Longgu-Muli.

In the present study, we decomposed CLM into three parts: XchD (including Chaihu, Huangqin, Banxia, Shengjiang, Renshen, and Dazao), Longgu-Muli (LM) and the remaining part of CLM (including Guizhi, Fuling and Dahuang, GFD) (Table 1). Subsequently, we compared the effect of XchD, LM+GFD, XchD+LM, and XchD+GFD. The dose of decomposed recipes was proportional to the dose of CLM. Calculation formula of decomposed recipes (D): D = A*dosage of CLM/B. A = weight of decomposed recipes (XchD, LM+GFD, XchD+LM, XchD+GFD); B = weight of CLM (Table 2).

**Table 2:** Dosage of CLM and its decomposed recipes in the experiment

Pricriptions	Weight of herbal medicine (g)	Dosage of herbal medicine (g/kg)	Dosage of the yield (g/kg)
LM	30.75	5	2.1
XchD	18.75	3	1.28
LM+GFD	12	1.95	0.82
XchD+LM	23.25	3.78	1.59
XchD+GFD	26.25	4.27	1.79

### HPLC analysis

**Figure 1:**
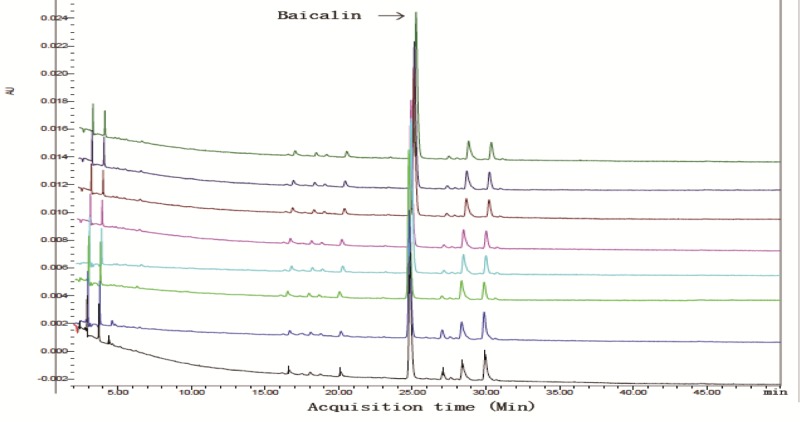
The quality control of different batches of CLM evaluated by HPLC Mobile phase: 0.5% Phosphoric acid solution (A) and Acetonitrile (B); centrifuged at 10000 rpm for 5 min. Gradient elution: 0–90 min, 90-10% A, 10-90% B; 90–92 min, 10-0% A, 90-100% B. The flow rate was 1 ml/min, working potential was 650 mV for the guard cell and 300 mV (E1) and −150 mV (E2) for the analytical cell and the column temperature was ambient. The injection volume was 10 μl, and the column temperature was maintained at 35°C. The DAD detector was set at 260 nm for acquiring chromatograms. Baiclin in CLM was tested by HPLC and used as a reference. Colors represent separated groups of herbs. The x-axis represents acquisition time (Min) and the y-axis indicates intensity (AU). As shown in this figure, there was a similar chromatographic pattern in different batches of CLM herbal solutions. This suggested a good reproducibility for this herbal medicine preparation.

The quality control of different batches of CLM was evaluated using HPLC method. The HPLC analysis was performed on a Waters 2695 Alliance HPLC system (Waters Corp., Milford, MA, U.S.A.). The raw data were detected with 2998 DAD and processed with Empower Software. An Apollo C_18_ column (250 mm*4.6 mm, 5 μm) preceded by a Waters Symmetry Shield RPC_18_ guard column (20 mm*3.9 mm, 5 μm) was applied for all analyses (Figure 1 and Table 3).

**Table 3:** Both peak area and concentration of baicalin in CLM by HPLC analysis

Sample	Concentration (mg/ml)	Peak area (AU)
Aicalin	0.028	103 ± 1
CLM	0.086	125 ± 1

### Behavioral tests

#### Open field test

The open field test (OFT) was conducted to assess the locomotor activity and exploratory behavior in an open area. The spontaneous locomotor activity was measured in a square arena (40 × 40 × 15 cm) for monitoring the horizontal activity (i.e. total distance traveled). Mice were tested in a well-illuminated (∼300 lux) transparent acrylic cage for 5 min. The mice activity was tracked near the bulkhead and in the central region of the arena. Distance (cm) and time spent in the central zone were analyzed. The arena was thoroughly cleaned between animals using 75% ethanol.

#### Tail suspension test

In a chamber, both acoustically and visually isolated, an individual mouse was suspended 50 cm above the floor by adhesive tape which was placed approximately 1 cm from the tip of the tail. Activities of the animals were videotaped. The computer calculated the total duration of immobility during the last 4 min of a 6-min testing session.

#### Forced swim test

The Forced swim test (FST), which is a widely used paradigm for behavioral despair and antidepressant response in rodents, was performed as reported previously [21]. Briefly, mice were removed from their home cages, placed individually into a clear glass tank (height: 40 cm; diameter: 20 cm) filled with 30 cm of water (22–23°C), and allowed to swim for 6 min [22]. The mice were considered immobile when floating in water without struggling and making only those movements necessary to keep their heads above the water surface. Total immobility time during the last 4 min of the 6-min testing session was recorded using ANY-maze software.

### Novelty-suppressed feeding

Mice were food-deprived for 24 h and subsequently placed into a 40 × 40 cm open field. A single pellet of the mice normal food chow was placed in the center of the open-field arena. Each animal was introduced into a corner of the arena and allowed to explore for up to 10 min. The trial ended when the mouse chewed a part of the chow. The amount of food consumed in the home cage was considered as the weight of chow consumed and was used as a control measure for appetite. The latency to begin eating in the open field, defined as active chewing of the pellet, was also recorded.

### Western blotting

The whole hippocampus (ventral and dorsal) was rapidly dissected, frozen, and lysed in buffer containing protease and phosphatase inhibitors. The total protein amount was quantitated using the Bradford method. Protein lysates were separated by 12% SDS/PAGE and were transferred on to PVDF membranes. BDNF quantitcation was carried out using SDS/PAGE. Primary antibodies against BDNF (1:200; Santa Cruz Biotechnology) and β-tubulin (1:2000; Cell Signaling) were used, while anti-rabbit secondary antibodies were used at 1:2000. The blots were visualized using the Super Signal West Pico Chemiluminescent Substrate (Thermo Fisher Scientific Inc.). The amount of BDNF was normalized to the β-tubulin bands. All experiments were performed in triplicates.

### Statistical analysis

Data were presented as the mean ± S.E.M. Statistical evaluation was performed using multiple comparisons made using one-way ANOVA, followed by the Newman–Keuls multiple range test, and a value of *P*<0.05 was considered statistically significant.

## Results

### Screening of the CLM dosage for rapid antidepressant potential

**Figure 2:**
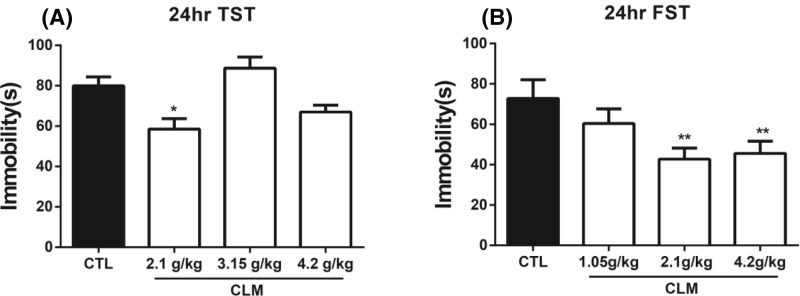
Effects of CLM on the TST and FST at different doses of CLM post a single administration (**A**) TST was carried out at 24 hr after CLM administration. (**B**) FST was carried out at 24 hr after CLM administration. Immobility time was measured for the last 4 min during the 6-min testing time in both TST and FST. Group: Control (CTL), CLM (1.05, 2.1, 3.15, 4.2 g/kg). *P<0.05, **P<0.01, compared with control. Statistical evaluation was performed using multiple comparisons made using one-way ANOVA, followed by the Newman–Keuls multiple range test, and a value of *P*<0.05 was considered statistically significant. Data represent mean ± S.E.M. and *n*=8/group.

To identify the effective dose of CLM with a rapid antidepressant-like potential, we first screened the dose that elicited a relatively long antidepressant effect in tail suspension test (TST) post a single administration. At 24 hr after a single administration of CLM with the dosages equivalent to or lower than clinical use (1.05, 2.1, 3.15, and 4.2 g/kg), there was significant treatment effect for both TST (one-way ANOVA, F _(3, 28)_ = 8.148, *P*<0.05, and *n*=8/group; Figure 2A) and FST (one-way ANOVA, F _(3, 28)_ = 4.943, *P*<0.01, and *n*=8/group; Figure 2B). Post-hoc analysis indicated that 2.1 g/kg reduced the immobility in both TST and FST. Although 4.2 g/kg reduced the immobility time in FST (*P*<0.01 compared with CTL), 3.15 or 4.2 g/kg did not have an effect on TST. Therefore, 2.1 g/kg, which is a half of equivalent clinical dose, was used for verification of the rapid antidepressant potential of CLM in the following tests.

### CLM elicited antidepressant effect immediately and persistently

**Figure 3:**
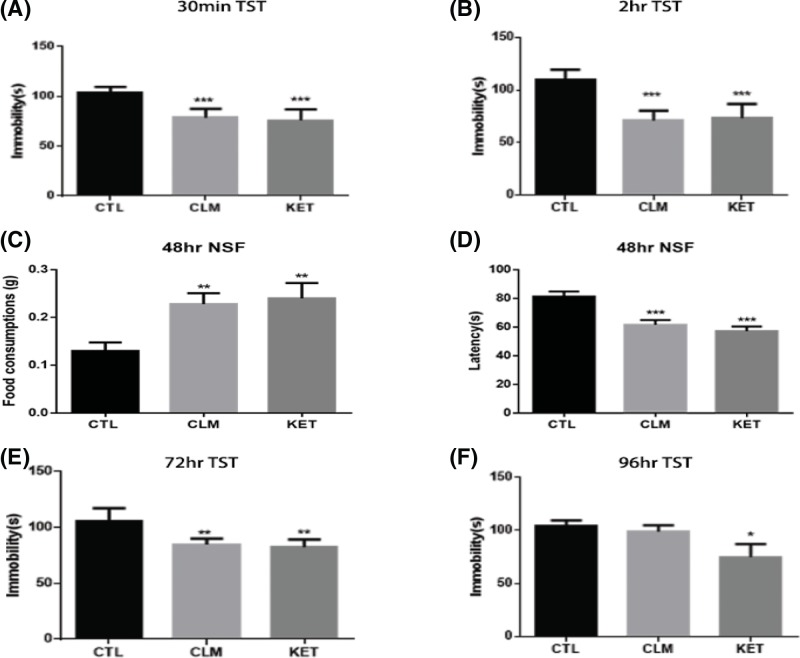
Effects of CLM on the TST and NSF at different times post single administration (**A**,**B**,**E**,**F**) TST was carried out at 30 min, 2, 72, and 96 hr post CLM and ketamine administration. Group: control (CTL), CLM (2.1 g/kg), KET (ketamine, 30 mg/kg). Immobility time was measured for the last 4 min during the 6-min testing time in TST. *P<0.05, **P<0.01, ***P<0.001 compared with control. (**C**,**D**) NSF test was carried out at 48 hr after CLM and ketamine administration. The time of latency to feed and the change in the total amount of food consumed were measured during 10-min test of NST. **P<0.01, ***P<0.001 compared with control. Statistical evaluation was performed using multiple comparisons made using one-way ANOVA, followed by the Newman–Keuls multiple range test, and a value of *P*<0.05 was considered statistically significant. Data represent mean ± S.E.M. and *n*=10/group.

The rapid antidepressant effects of CLM were examined over time following a single-dose administration, using ketamine as a positive control. To avoid the effects of repeated testing, only naïve animals were used at different time points. There was a significant effect of treatment for TST at 30 min (one-way ANOVA, F _(2, 27)_ = 21.6, *P*<0.001, and *n*=10/group; Figure 3A), 2 hr (one-way ANOVA, F _(2, 27)_ = 27.8, *P*<0.001, and *n*=10/group; Figure 3B); 72 hr (one-way ANOVA, F _(2, 27)_ = 8.708, *P*<0.01, and *n*=10/group; Figure 3E), and 96 hr (one-way ANOVA, F _(2, 27)_ = 3.44, *P*<0.05, and *n*=10/group; Figure 3F). Post-hoc analysis indicated that CLM reduced immobility in TST from 30 min to 72 hr, whereas the antidepressant action of ketamine lasted for at least 96 hr. The novelty-suppressed feeding (NSF) test is commonly designed to assess the anxiolytic and/or antidepressant effects following treatment with chronic, but not acute or subchronic SSRIs in rodents [23,24], thus suitable to test the rapid antidepressant efficacy post an acute administration. Forty-eight hours after the single dose of CLM or ketamine, the latency to feed was reduced significantly (one-way ANOVA, F _(2, 27)_ = 14.10, *P*<0.001, and *n*=10/group; Figure 3C), and food consumption increased in NSF (one-way ANOVA, F _(2, 27)_ = 5.833, *P*<0.01, and *n*=10/group; Figure 3D). Post-hoc analysis indicated that CLM and ketamine reduced the latency and increased food consumption.

### CLM did not affect the locomotor activity in the OFT

**Figure 4:**
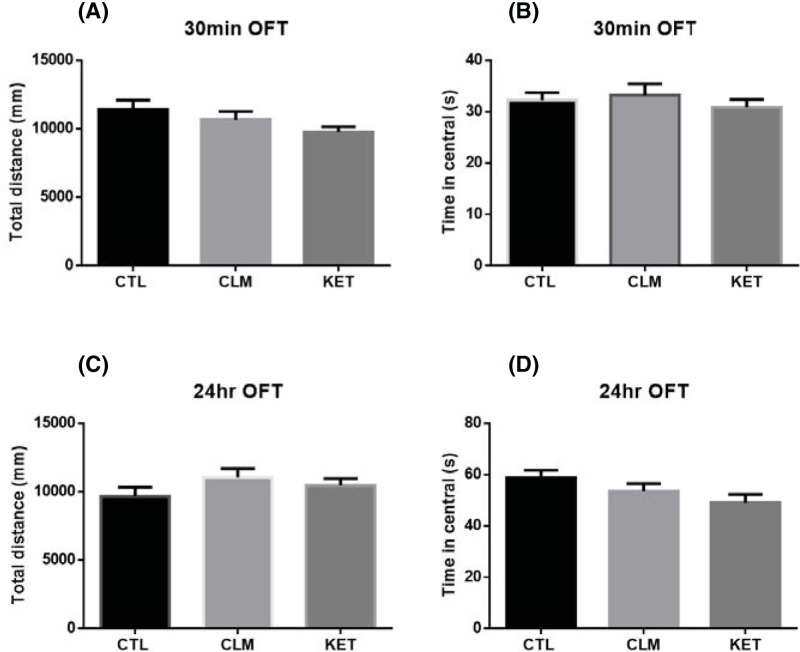
Effects of CLM on the OFT Mice were grouped as CTL (saline i.g), CLM (2.1 g/kg, i.g), KET (30 mg/kg, i.p) and tested at 30 min and 24 hr post administration. (**A**) Total distance traveled during a 5-min open field testing time at 30 min after CLM and ketamine administration, *P*=0.146. (**B**) Time spent on the center part during a 5-min open field testing time at 30 min after CLM and ketamine administration, *P*=0.647. (**C**) Total distance traveled during a 5-min open field testing time at 24 hr after CLM and ketamine administration, *P*=0.269. (**D**) Time spent on the center part during a 5-min open field testing time at 24 hr after CLM and ketamine administration, *P*=0.098. Values represent mean ± S.E.M. Statistical evaluation was performed using multiple comparisons made using one-way ANOVA, followed by the Newman–Keuls multiple range test, and a value of *P*<0.05 was considered statistically significant. *n*=10/group.

The OFT estimated locomotor activity and anxiety-like behavior. Both CLM and ketamine at their effective doses did not have significant effects on either the locomotor activity (total distance) or the time spent in the center of the open-field arena at either 30 min or 24 hr post CLM: for total distance traveled during a 5-min open field testing time (30 min: ANOVA, F _(2, 27)_ = 2.066, *P*=0.146, and *n*=10/group; 24 hr: ANOVA, F _(2, 27)_ = 1.38, *P*=0.269, and *n*=10/group, Figure 4A,C); for time spent on the center zone (30 min: ANOVA, F _(2, 27)_ = 0.443, *P*=0.647, and *n*=10/group; 24 hr: ANOVA, F _(2, 27)_ = 2.534, *P*=0.098, and *n*=10/group, Figure 4B,D).

### XchD was primarily responsible for rapid antidepressant-like effects of CLM

**Figure 5:**
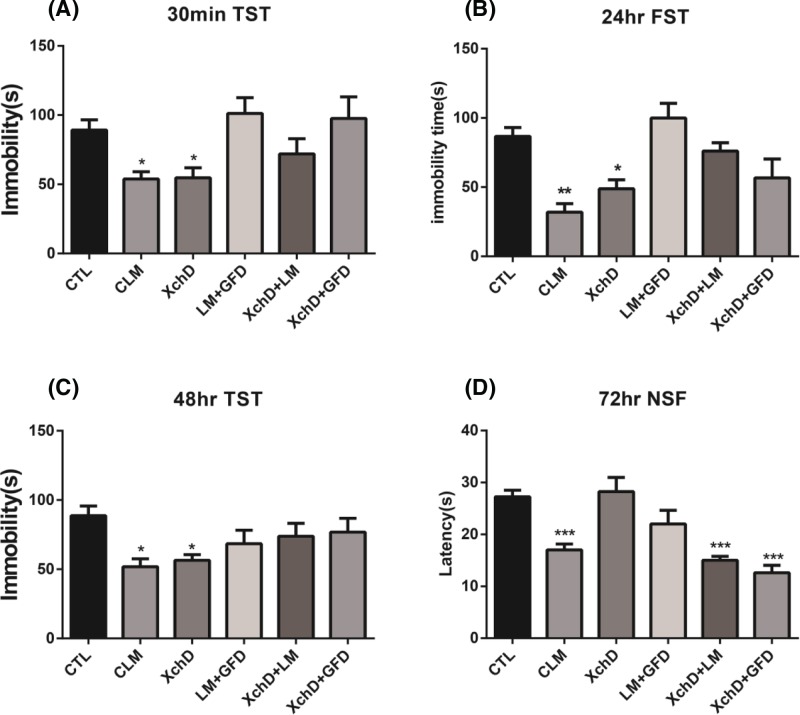
XchD was primarily responsible for rapid antidepressant-like effect of CLM Mice were grouped into CTL, CLM, XchD, LM+GFD, XchD+LM, XchD+GFD. (**A**,**C**) TST was carried out at 30 min and 48 hr after drug administration. (**B**) FST was carried out at 24 hr after drug administration. Immobility time was measured for the last 4 min during the 6-min testing time in both TST and FST. Compared with control group, a single administration of both CLM and XchD reduced the immobility time in TST at 30 min and 48 hr. By 24 hr, CLM and XchD significantly reduced the immobility time in FST. (**D**) NSF test was carried out at 72 hr after drug administration. The time of latency to feed was measured during 10-min test after a single administration. XchD did not reduce the latency to feed in NSF, whereas both XchD+LM and XchD+GFD, similar to CLM significantly reduced the latency to feed. *P<0.05, **P<0.01, ***P<0.001 compared with control. Statistical evaluation was performed using multiple comparisons made using one-way ANOVA, followed by the Newman–Keuls multiple range test, and a value of *P*<0.05 was considered statistically significant. Data represent mean ± S.E.M. and *n*=6–8/group.

Based on the formulation rule, CLM is a composite formula which derives from XchD, together with other constituents including the sedative component LM and GFD. We then tested if XchD played a major role in the rapid antidepressant-like effect of CLM, and how XchD interacted with the other parts. We compared the antidepressant response across groups of CLM, XchD, LM+GFD, XchD+LM, and XchD+GFD, each with dose proportionally in the whole formula of CLM. There were significant treatment effects for TST (one-way ANOVA, F _(5, 51)_ = 4.488, *P*<0.01, and *n*=6–8/group; Figure 5A) at 30 min and 48 hr (one-way ANOVA, F _(5, 43)_ = 2.682, *P*<0.05, and *n*=6–8/group; Figure 5C). Similar effects were also found for FST (one-way ANOVA, F _(5, 36)_ = 8.505, *P*<0.001, and *n*=6–8/group; Figure 5B) at 24 hr, and NSF (one-way ANOVA, F _(5, 33)_ = 8.247, *P*<0.001, and *n*=6–8/group; Figure 5D) at 72 hr. Post-hoc analysis indicated that CLM reduced both the immobility time in TST and FST and the latency to eat in NSF at these time points. Amongst the three major constituents, only XchD significantly reduced the immobility time in TST at 30 min (*P*<0.05 compared with CTL), which remained to be effective at 48 hr (*P*<0.05 compared with CTL). By 24 hr, XchD also demonstrated the antidepressant effect in FST (*P*<0.05 compared with CTL), consistent with CLM (*P*<0.01 compared with CTL). Interestingly, at 72 hr, there was no effect of XchD on reducing the latency to eat in NSF (*P*>0.05 compared with CTL), whereas both XchD+LM (*P*<0.001 compared with CTL) and XchD+GFD (*P*<0.001 compared with CTL), showed the significant effect similar to CLM (*P*<0.001 compared with CTL). Post-hoc analysis indicated that CLM, XchD+LM, and XchD+GFD reduced the latency at NSF 72 hr post single administration. Together these observations not only demonstrated the essential role of XchD in eliciting antidepressant effect of CLM, but also indicated other components, had important supporting role in maintaining the effect.

### BDNF expression increased at 30 min and 24 hr post a single dose of CLM

**Figure 6:**
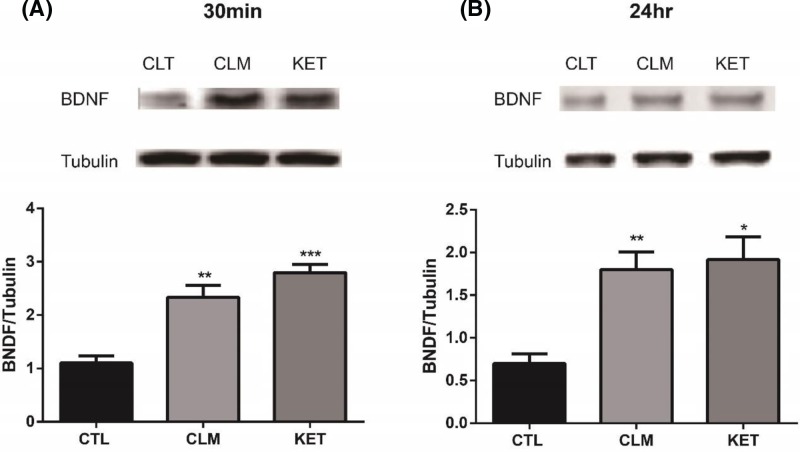
BDNF expressions in the hippocampus over time post an acute administration of CLM (**A**) BDNF protein expression levels in mouse hippocampus were determined by Western blot at 30 min after treatment with CLM or ketamine (KET). At 30 min, BDNF increased significantly after a single administration of CLM and ketamine. (**B**) BDNF protein expression levels in mouse hippocampus were determined by Western blot at 24 hr after drug treatment. At 24 hr, it showed a similar effect. BDNF expression significantly increased from 30 min to 24 hr after a single administration of CLM and ketamine. Group: control (CTL), CLM (2.1 g/kg), KET (ketamine, 30 mg/kg). *P<0.05, **P<0.01, ***P<0.001 compared with control. Statistical evaluation was performed using multiple comparisons made using one-way ANOVA, followed by the Newman–Keuls multiple range test, and a value of *P*<0.05 was considered statistically significant. Data represent mean ± S.E.M. and *n*=6/group.

The instant expression of protein level of BDNF has been previously associated with the rapid antidepressant effect of ketamine and herb medicine Yueju [19]. To understand the molecular mechanisms underlying the antidepressant effects of CLM, we examined the expression of BDNF in the hippocampus at 30 min and 24 hr following CLM or ketamine administration. The results indicated that BDNF in hippocampus significantly increased 30 min (one-way ANOVA, F _(2, 15)_ = 25.4, *P*<0.001, and *n*=6/group; Figure 6A) and 24 hr (one-way ANOVA, F _(2, 15)_ = 10.7, *P*<0.01, and *n*=6/group; Figure 6B) post a single administration.

## Discussion

To date, depression treatment with rapid onset is still a significant challenge. CLM has been long-time safely and effectively used in patients with many conditions including depression, and here we further assessed whether it had rapid antidepressant effects and underlying mechanisms. In the current study, a single dose of CLM instantly induced immediate antidepressant-like effect in the NSF and TST tests. Analysis of the constituents of XchD and other parts of CLM indicated XchD was essential for CLM’s immediate and persistent antidepressant effect, whereas other parts had important supporting role in maintaining the antidepressant effect for a longer time. Furthermore, a single dose of CLM immediately increased the expression of BDNF in the hippocampus, which is likely associated with the rapid antidepressant-like effect of CLM.

The present results indicated that CLM has a great potential as a fast-onset antidepressant. TST is a widely used behavioral paradigm for assessing the effects of antidepressants in animal models of depression [25], and here the performance of TST at different time points indicated antidepressant effects of CLM from 30 min to 72 hr. NSF has been well accepted to be used for testing fast-onset antidepressant effect following a single administration [26]. A single dose of CLM resulted in improvement in latency to eat and food consumption in the NSF test, supporting the fast-onset nature of the antidepressant effect of CLM.

The immediate antidepressant activity of CLM was comparable with Yueju pill or ketamine, but the effect of CLM did not last for 5 days as Yueju or ketamine [19,20]. It is, however, worth noting that the dose of CLM to induce rapid and lasting antidepressant effects was only half of the clinical dose. In contrast, higher than clinical dose was required for Yueju pill to elicit rapid antidepressant-like effects [19], whereas we found a single full clinical dose of Yueju only induced a shorter duration of antidepressant activity [27]. Importantly, we determined experimentally an optimal dose of CLM for rapid antidepressant-like response, which is consistent with the two half doses of prescription of the herb preparation for daily use in humans.

The present findings also provided the experimental evidence to elucidate the role of XchD in antidepressant effect of CLM. We found XchD was required for the immediate antidepressant response, but the duration of the activity was shortened without other parts of CLM. Both LM and GFD prolonged the effect of XchD. These results demonstrate the value of combination of different parts for Chinese herbal medicine. Baicalin, a major active flavonoid compound in *Scutellaria baicalensis* (Huang Qin) in CLM, possesses multiple pharmacological activities such as anti-inflammatory, and promoting neurogenesis capabilities [28,29], and showed an antidepressant-like effect [30,31], related to the increase in BDNF in the hippocampus [28,32]. In the present study, baicalin was the most enriched compound in the CLM preparation, contributed by Huangqin in the XchD, raising the possibility that baicalin may play some part in rapid antidepressant efficacy of CLM. However, further study is warranted to clarify this issue.

Deficiencies in neuroplasticity are pathogenesis of depression [33], and neuroplasticity has been held as the most important therapeutic target of antidepressants. Increased BDNF translation is suggested to play an important role in regulating the rapid antidepressant effects of ketamine and Yueju [8]. The present study showed the BDNF up-regulation at 30 min and 24 h, which was likely responsible for its immediate and relatively lasting antidepressant activity. Therefore, the increase in BDNF in hippocampus may play a key role for the rapid antidepressant-like effect of CLM, similar to ketamine.

## Abbreviations

BDNF: brain-derived neurotrophic factor
CLM: Chaihu-jia-Longgu-Muli-tang
FST: forced swim test
GFD: Guizhi, Fuling and Dahuang
LM: Longgu-Muli
NSF: novelty-suppressed feeding
OFT: open field test
SSRI: selective serotonin reuptake inhibitor
TCM: traditional Chinese medicine
TST: tail suspension test
XchD: Xiaochaihu decoction
